# Mineral inclusions in diamonds may be synchronous but not syngenetic

**DOI:** 10.1038/ncomms14168

**Published:** 2017-01-24

**Authors:** Fabrizio Nestola, Haemyeong Jung, Lawrence A. Taylor

**Affiliations:** 1 Dipartimento di Geoscienze, Università degli Studi di Padova, Via G. Gradenigo 6, 35131 Padova, Italy; 2 School of Earth and Environmental Sciences, Seoul National University, 151-747 Seoul, Korea; 3 Department of Earth and Planetary Sciences, University of Tennessee, Knoxville, Tennessee 37996-1410, USA

## Abstract

It is widely assumed that mineral inclusions and their host diamonds are ‘syngenetic' in origin, which means that they formed simultaneously and from the same chemical processes. Mineral inclusions that, instead, were formed earlier with respect to diamonds are termed protogenetic. However, minerals can have the same age as the diamonds in that they become enclosed in and isolated from any further isotopic exchange. But this is termed ‘synchronous' not ‘syngenetic'. Here we demonstrate conclusively the protogenesis of inclusions in diamonds, based upon data from an exceptional fragment of a diamond-bearing peridotite, its clinopyroxene and a gem-quality diamond. Clinopyroxenes in the xenolith had the same chemistry and crystallographic orientation as those for inclusions in the diamond. With our results with garnets, olivines and sulfides, we can state that a major portion of the mineral inclusions in non-coated, monocrystalline-lithospheric diamonds are protogenetic. Our discovery here presented has implications for all genetic aspects of diamond growth, including their ages.

## Abstract (web summary)

Trapped mineral inclusions in diamonds give information on diamond crystallization and ages, under the assumption that they are syngenetic (formed simultaneously). Here, the authors show evidence that many mineral inclusions are protogenetic (formed at different times) thus undermining previous diamond ages.

Diamonds and their mineral inclusions are among the most intriguing natural samples on Earth, as they represent a unique opportunity to probe the deepest regions of our planet. Indeed, diamonds are thought to be among the oldest available materials—the oldest diamonds have been dated at up to 3.5 billion years^1^,^2^,^3^,^4^,^5^). In addition, diamond is also able to capture mineral inclusions at depths from even greater than 500 km^6^,^7^. Such a combination makes diamond and its pristine mineral inclusions a virtual ‘window into the Earth's mantle', providing crucial information about the geological evolution of our planet. However, it is also well known and appreciated that the age and crystallization environment of diamond are only based upon its mineral inclusions; indeed, the age of diamonds are determined by dating the mineral inclusions trapped within them; similarly, the depth of diamond formation is determined by studying the depth of formation of the inclusions^8^,^9^,^10^.

The validity of the criteria for the ‘syngenetic' co-crystallization of diamonds and their inclusions is at the very foundation of all diamond inclusion studies. Virtually every paper on mineral inclusions in diamonds is based upon an essential assumption that the mineral inclusions and their host diamond are syngenetic—that is, formed simultaneously and from the same formational process. However, supporting evidence for this assumption is rarely presented. There have been an extensive series of papers written with reviews of the criteria for ‘syngenesis' of mineral inclusions and their host diamonds^11^,^12^,^13^,^14^, each coming to the conclusion that most, if not all, diamonds and their inclusions are syngenetic. Others believe the opposite—that most, if not all, inclusions in diamonds are protogenetic—formed before encapsulation in the diamonds^15^,^16^,^17^,^18^,^19^,^20^,^21^,^22^,^23^,^24^. The syngenesis versus protogenesis relationship between diamond and its mineral inclusions represents an extended scientific debate in diamond research; it is likely that the many decade-years old debate has not been well-addressed, at least, until recently. In general, we can count only a few publications where protogenesis is really invoked^15^,^16^,^17^,^18^,^19^,^20^,^21^,^22^,^23^,^24^. This illustrates how crucial the principle of ‘syngenesis' is in diamond research. We will address the criteria for establishing the syngenesis of diamond and its mineral inclusions. This will be placed within the context of the evidence, presented in this paper, as well as in prior studies, that most mineral inclusions in diamonds are simply enclosed, pre-existing minerals—that is, protogenetic—and are synchronous, not syngenetic.

In this study, based upon the morphology, crystallography and chemistry of mineral inclusions in non-coated, monocrystalline-lithospheric diamonds, we present unequivocal evidence for the protogenesis of these inclusions, not syngenesis. In addition, we address the popular use of the term ‘syngenesis' for such inclusions. Some mineral inclusions may be ‘synchronous' (e.g., monosulfide solid solution); however, virtually no silicate inclusions are truly syngeneous—representing the same radiogenic isotopic ages. However, the possibility of syngenesis can still permit the age presented by the mineral inclusion to represent the same age as its diamond host.

## Results

### Syngenesis versus protogenesis and the case of diopsid

The two main arguments in favour of syngenesis are focused on the morphology of the inclusions, which often show a pseudocubo-octahedral morphology imposed by the diamond^11^,^25^,^26^, and on the assumption that diamond and its mineral inclusions show epitaxial growth relationships (coincidence of crystallographic planes and directions between the diamond and its inclusions^12^,^13^,^14^,^27^). There are other arguments based upon C and O isotopes, in coexisting diamond and silicate inclusions, which have been made by Ickert *et al*.^28^, but this approach is a subject of considerable debate^29^ and will not be addressed further.

Relative to the inclusion morphologies, recent studies^24^,^30^,^31^ reported strong evidences for protogenesis of olivines included in diamonds from the Siberian (Russia) and Kaapvaal (South Africa) cratons. This is in spite of the olivines having pseudocubo-octahedral morphology. Indeed, the cubo-octahedral imposed morphology by diamond is extremely common for all types of inclusions and such a morphology has always been considered a strong argument in favour of syngenesis. However, this cubic super-imposed morphology, reported to indicate simultaneous crystallization from same fluid at the same time^13^, has no experimental and conclusive proof of this contention in spite of continued reference to Sunagawa^32^,^33^.

**Figure 1:**
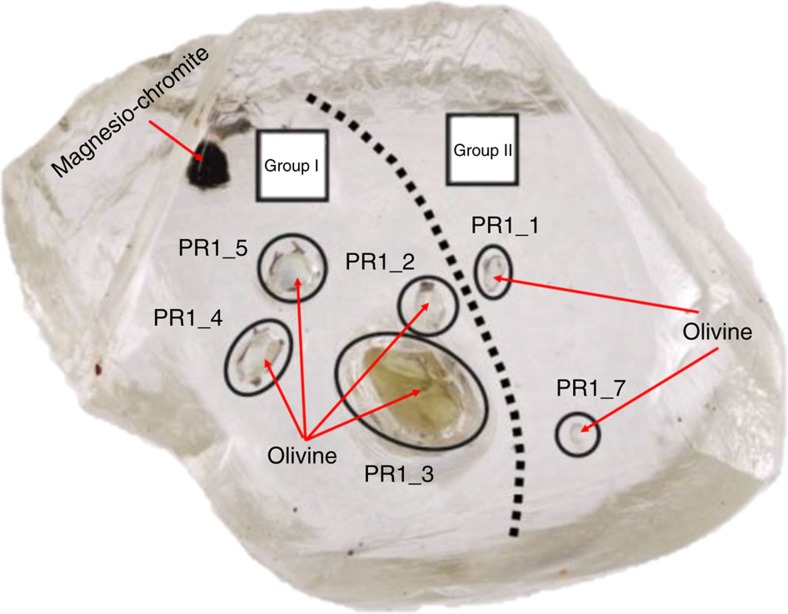
A gem-quality octahedral diamond from the Kaapvaal craton in South Africa. The diamond shows seven different mineral inclusions, modified after Milani *et al*.^31^. Six inclusions are olivines and the seventh one is a crystal of magnesio-chromite. The six inclusions of olivine belong to two different groups, group I and group II, respectively. Each group shows identical crystallographic orientation. The different colour of inclusion PR1-3 is likely due to an optical effect, due to a greater thickness. The inclusions, indeed, have all the same chemical compositions, as demonstrated by the identical unit-cell parameters^31^.

**Figure 2:**
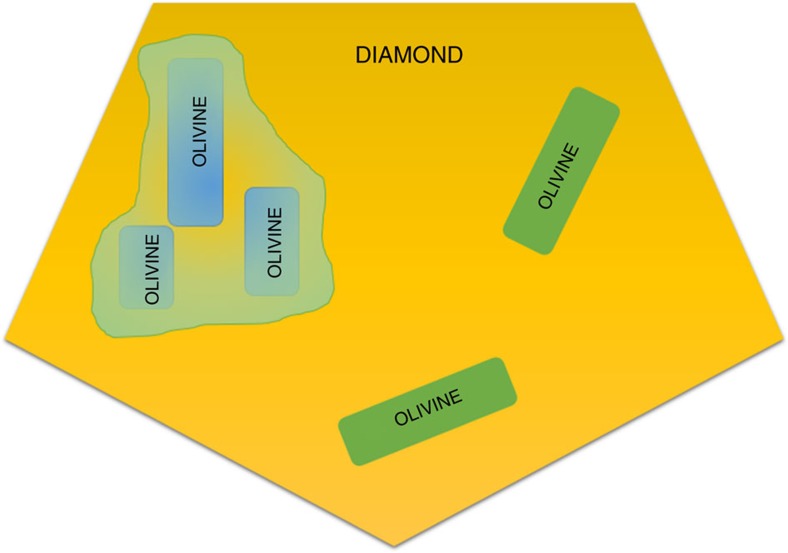
Possible diamond–olivine growth mechanism. The cartoon in the figure shows a possible diamond–olivine growth relationship, as proposed by and modified from previous studies^24^,^30^. The host diamond is in yellow; the shapes of the olivine crystals used in this cartoon are not real but are simplified. The cartoon explains the experimental observations from different works in which multiple inclusions of olivines show different crystallographic orientations but at the same time, in the same diamond, it is also possible to find different olivines with similar crystallographic orientation. This orientational similarity can be obtained only if these olivines belonged to the same pre-existing olivine monocrystal. This is strong evidence of olivine protogenesis.

Relatively to the reported ‘epitaxy' of diamond and its inclusionsrecent studies^24^,^31^ by single-crystal X-ray diffraction definitively showed that no epitaxial relationship between olivine and diamond was discovered on a huge population of inclusions in diamonds from different localities. Indeed, one of the most interesting discoveries, relative to olivine inclusions, is that they are totally randomly oriented within their diamond host. However, in single diamonds, with multiple olivine inclusions, some olivine crystals have an identical crystallographic orientation to each other^24^,^31^, yet not to their diamond hosts, as shown in Fig. 1 and represented in a cartoon in Fig. 2. This experimental evidence was interpreted as the main argument in favour of the protogenetic origin of such olivines, with respect their diamond hosts. The explanation for this conclusion was that the iso-oriented crystals could be only interpreted as the remaining ‘islands' of a pre-existing olivine mono-crystal after a resorption process involved in the diamond crystallization. Additional experimental evidence for protogenesis is statistical in that of all the diamond hosts and the inclusions investigated (28 diamonds and 63 olivine inclusions), the authors^24^,^31^ could not find a single diamond with any set of crystallographic orientations in common with the olivine inclusion. Indeed, some diamonds contained up to seven olivine inclusions. Their interpretation was explained in terms of the crystallization of the diamond, which would be energetically favoured at a triple junction between pre-existing olivines in the peridotite host-rock, during introduction of the metasomatic fluid that formed the diamond. However, this is only an interpretation, and until the present study, quantitative evidence has been lacking to demonstrate it.

### Evidence of protogenesis

**Figure 3:**
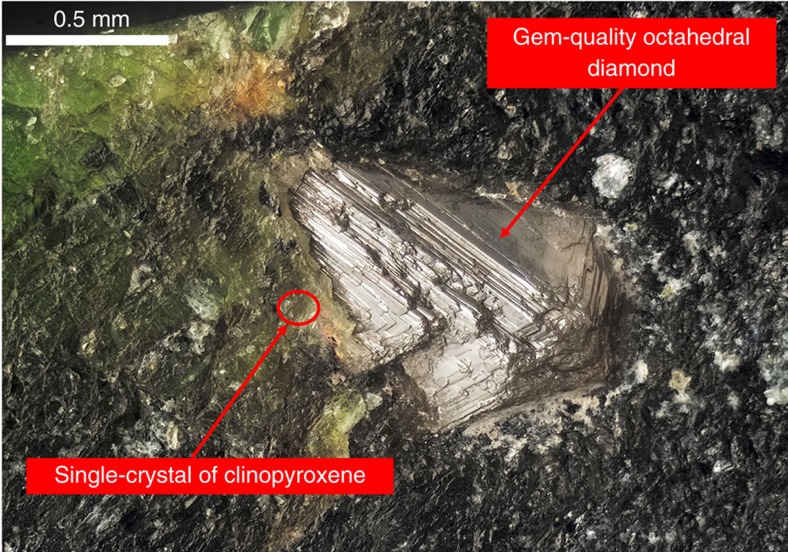
Exceptional fragment of diamond-bearing peridotitic rock. This exceptional rock fragment, with a super-imposed gem-quality, millimetre-sized diamond, contains olivine and clinopyroxene inclusions identical in chemistry and crystallographic orientation to those outside the diamond, a clear evidence for protogenesis of the olivine inclusions, with a genesis similar that is illustrated in Fig. 2.

We have investigated, by single-crystal X-ray diffraction (hereafter XRD), a coarse-grained diamond-bearing garnet peridotite xenolith from the Finsch mine (Kaapvaal craton, South Africa). This peridotite contains a large millimetre-sized, gem-quality, octahedral diamond, still embedded in its host rock. This portion of the xenolith comes from the same sample^34^ and is shown in Fig. 3. The gem-quality octahedral diamond is well visible with its vertex pointing away from the image and is embedded in a matrix of serpentine, orthopyroxene, garnet and clinopyroxene. Owing to the intersection of the planar-growth planes, and its position with the majority of the diamond below the surface, it was not possible to detect any inclusions with a binocular microscopy. However, with the efficient use of a prototype, XRD instrument (see Methods), it was possible to perform a complete XRD scan of the internal part of the diamond, without extracting it from the host rock; this resulted in the detection and identification of two different inclusions, one an olivine and the other a peridotitic clinopyroxene. The experimental technique adopted in this work permits determination of the orientation matrixes of the two inclusions, as well as that of the diamond. In this manner, it was possible to determine reciprocal crystallographic orientations of the inclusions–diamond pairs^24^,^31^. The two inclusions (i.e., olivine and clinopyroxene) do not show any specific crystallographic orientation relative to each other, and importantly, they do not have any orientational relationships with the host diamond (see Table 1). Based upon prior detailed crystallographic studies, this result was expected for the diamond–olivine pair^24^,^31^. We are not familiar with any previous study of diamond–clinopyroxene orientation relationships. However, the presence of a clinopyroxene-bearing diamond embedded in a rock matrix containing a clinopyroxene presents yet another intriguing possibility.

The presence of a single crystal of clinopyroxene positioned externally to the diamond is shown in Fig. 3. This ‘groundmass' clinopyroxene is set within a matrix of serpentine alteration and is located at around 0.1 mm from the external surface of the diamond. As evident in Fig. 3, the clinopyroxene crystal is not optically distinguishable. However, XRD analysis definitively identified this external clinopyroxene. Unexpectedly, the crystallographic orientation of this groundmass clinopyroxene outside the diamond is identical to the clinopyroxene included within the diamond. Minor angular mismatches between the crystallographic axes are only 0.8°, 0.6° and 1.6° degrees, for *a*, *b* and *c* axes, respectively (see Table 1); these angular values are within one experimental uncertainty. This means that the two crystals have an identical crystallographic orientation—that is, clinopyroxene outside and inside the diamond are, in our interpretation, part of one crystal. The significance of this observation is paramount to the question of syngenetic versus protogenetic diamond mineral inclusions, at least for non-gem-quality, coated, poly-crystalline diamonds, for which different interpretations have been reported^35^,^36^,^37^,^38^.

The surprising first experimental confirmation of co-incidence of the crystallographic orientation of the pyroxene outside and inside a diamond was effectively predicted as ‘remaining islands' of pre-existing mono-crystals^24^. The significance of this observation is monumental to the genetic relations between diamond and its mineral inclusions. These exciting results from our present study indicate that such inclusions are definitively protogenetic. This is ‘proof-positive' for protogenesis for the diamond and its inclusion.

## Discussion

Formation age of diamonds is determined by measurements of their mineral inclusions: K–Ar in pyroxene, only reliable for dating the kimberlite; Rb–Sr and/or Sm–Nd in garnet and pyroxene; and Re–Os and U–Th–Pb in sulfide phases. The application of the Sm–Nd and Rb–Sr isotopic schemes, due to limiting mineral masses necessary for instrumental sensitivity, requires putting together literally hundreds of inclusions from hundreds of diamonds, whereas the sulfide isotopic schemes permit ages on single diamonds. To date, the most commonly used and agreed upon mineral system is the Re–Os of sulfide inclusions. However, the major system that has received the most attention, until recently, is that of the Sm–Nd in garnet and pyroxene^39^,^40^,^41^.

The Sm–Nd system for age-dating is restricted for use if an isochron can be constructed from two or more minerals in isotopic equilibrium or one mineral and its corresponding whole rock. A major assumption is thereby made that the garnets and clinopyroxenes in the host rocks were in diffusional equilibrium and encapsulated above their ‘closure temperatures' (*T*_C_≈600–800 °C) for these radiogenic isotopes. This isolation effectively ‘freezes-in' the exchange of isotopic components, and starts their radiogenic clocks. However, for this system, the minerals in the host rock can continue to isotopically inter-diffuse (of course only when they are touching, many parageneses are non-touching), thereby re-equilibrating until they reach their *T*_C_—usually the date of kimberlite eruption^42^. The key limitations on the application of isotopic decay pairs are the availability and size of the inclusions, the abundance levels of the radionuclides and instrumental sensitivity.

The main assumption for radiogenic isotopic age determinations for diamonds, as well as rocks and minerals, is that they reach conditions where the minerals have stopped ‘isotopic communication' (diffusion) with their host rock and each other. As mentioned above, this can occur when they reach their isotopic closure temperature (*T*_C_); or when they are otherwise isolated from further isotopic diffusion with each other and their surroundings—for example, encapsulated in a diamond. All minerals are encapsulated in diamonds above their *T*_C_. In this encapsulation case, the minerals that are used for their radiogenic isotopes must have been the same ones in contact before isolation—for example, clinopyroxene and garnet. Therein is one of the major assumptions in age-dating of diamonds. Were the minerals that are being used in the isotopic-partitioning studies actually in contact prior to and during their encapsulation by the diamonds? Or, did they become diffusionally isolated while still in the rock, before encapsulation? In the case of a single-phase isotopic system, the encapsulation starts the isotopic system at a time=zero. This is the case for sulfide inclusions for Re–Os age determinations^2^,^3^,^4^,^5^, where the encapsulated immiscible-sulfide phase^1^ cools to an assemblage of pentlandite, pyrrhotite and chalcopyrite, and is isolated from any outside inter-action.

Landmark publications^39^,^43^ used the assemblage of garnet and clinopyroxene occurring as separate inclusions in diamonds—that is, non-touching=no elemental isotopic exchanges. Because there was a minimum mass necessary for instrumental sensitivity for accurate measurement of the isotopes in the minerals, literally hundreds of each mineral inclusion, from hundreds of diamonds, were accumulated together for the elaborate chemistry necessary for the isotopes—typically Sm–Nd and Rb–Sr—of the garnet and the clinopyroxene aggregations. In later studies^40^, the garnets were further separated into individual groups, depending upon colour—four in this specific study. They then only used the clinopyroxene and one of the four garnet groups for determining the diamond age, picking the garnet aggregation with the highest ^143^Nd/^144^Nd contents, and using the other three garnet groups to calculate the precision. The inherent assumption made in these studies is that all the diamonds formed instantaneously from a distinct event, and throughout the entire kimberlite. But the huge array of compositions for the garnets and clinopyroxenes testifies to a large variation in chemistry and time for the encapsulation into the diamonds.

**Figure 4:**
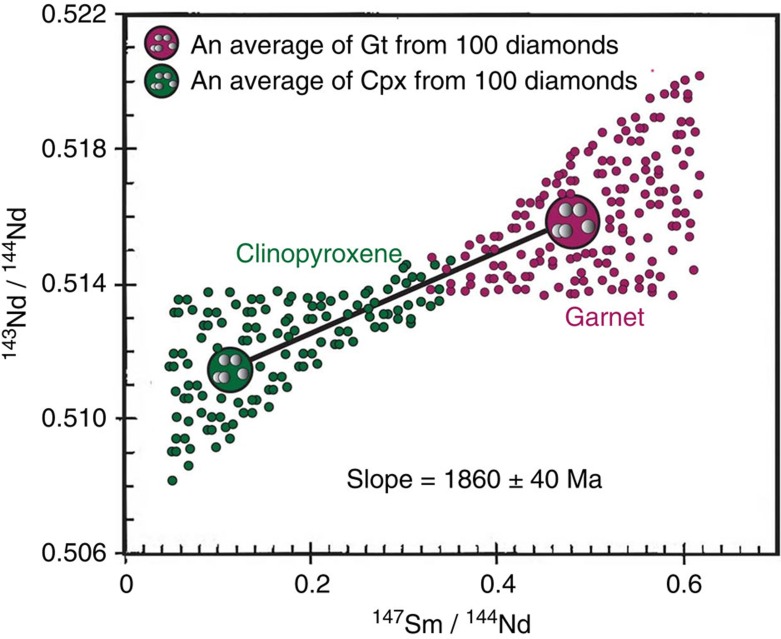
Sm–Nd two-mineral isochrons. Based upon the discussion by Navon^44^, and with modifications, the principles of this graph caution against the usage of multitudes of diamond inclusions for dating of ‘one diamond event'. It demonstrates that the nature of the two-mineral isochron is obviously a ‘grossly weighted-average age' and should only to be addressed and used with caution.

A modification of the principles presented by Navon^44^ on the formation of diamonds is shown in Fig. 4, based upon the criticisms of studies that were conducted of 100s of garnets and clinopyroxenes for diamond age determinations. This addresses the mis-conception of using piles of mineral inclusions for such conclusions. It is our contention that the assumption is false that these hundreds of garnet and clinopyroxene inclusion grains are all represented by one equilibrated pair of minerals, yet in an entire kimberlite. In reality, the diamond formation occurred over 10 to 100 s of million years, and such Sm–Nd two-mineral isochrons^39^ give but some indication of an ‘average diamond age', with large values of variability, and the acceptance of assumptions mentioned above. Such data were the best available at that time. However, in our opinion, the authors oversimplified the complications intrinsic to such practice. Abundant studies have shown that multiple silicate inclusions in diamonds represent multi-generations of diamonds that have experienced different metasomatic alterations^18^,^20^.

In summary, for the last couple of decades, culminating in the present study, mounting evidences have accrued that most, if not all, mineral inclusions are not syngenetic, but are protogenetic—that is, formed before the diamonds, perhaps billions of years before^18^,^19^,^20^. Several authors^18^,^20^,^45^ have presented evidence that questions and is considered to negate the assumption that the inclusions in diamonds from one kimberlite are of the similar composition or forming at the same time in isotopic equilibrium (syngenetic); this negated assumption is evidenced even within one xenolith, or even within one-single diamond. This was based upon some of the first diamondiferous eclogite tomography, with up to 78 macrodiamonds in one 65 g xenolith; each diamond was mapped as to its position relative to the others, and each diamond then examined and polished to reveal 1–6 mineral inclusions *in situ* on one polished diamond surface. Electron microprobe and secondary ion mass spectrometry (SIMS) analyses of the clinopyroxene inclusions in one diamond, for example, showed distinct chemical differences, including positive Eu anomalies. In fact, the clinopyroxene and garnet compositions in many diamonds possess inter-granular heterogeneities—they were completely different, gain to grain. Indeed, some authors^15^ examined multiple inclusions in one diamond; the 35 individual garnets inclusions in one diamond had compositions that covered the wide array of all eclogitic garnets from xenoliths recovered from kimberlites in Yakutia—all in one diamond. The additional five clinopyroxenes from this single diamond also exhibited inter-granular heterogeneities. Recently^21^,^46^, based upon water in olivine, pyroxene and garnets as diamond inclusions, caution was again presented for assuming syngenesis of simultaneous formation of diamonds and their inclusions.

The present study may help negate the use of ‘syngenesis' for mineral inclusions in diamonds. This term has been used for the formation of the inclusion and its host diamond at the same instant and from the same formational process. This is the incorrect word for use with respect to diamond mineral inclusions. The isotopic systems of immiscible sulfide phases can be started by the isolation of encapsulation, thereby resulting in the inclusions giving the time of encapsulation. This is synchronous. However, to assume that pairs of silicate minerals were touching or otherwise in isotopic equilibrium before encapsulation, and give synchronous ages, is incorrect. The age of the diamond and that of the inclusions may be synchronous, even if the inclusions existed before the encapsulation, but they are not syngenetic in origin.

## Methods

### Single-crystal XRD

The identification of the minerals investigated in this work and the determination of the orientation matrixes of diamond and its mineral inclusions, in addition to the mineral external to the diamond, were made possible with a prototype instrument at the Department of Geosciences at the University of Padova. The instrument is a single-crystal X-ray diffractometer Supernova (Rigaku-Oxford Diffraction) equipped with a brilliant X-ray micro-source (X-ray radiation wavelength=0.71073 Å; spot-size at the sample=0.12 mm) and with the Pilatus 200K detector (Dectris). Such an instrument permits measurement of small crystals down to 5 μm^47^. The orientation matrixes were then treated by OrientXplot software^48^, which allows one to calculate easily the reciprocal crystallographic orientations of the diamond-inclusions system.

### Data availability

All data generated or analysed during this study are included in this published article and incorporated in Table 1.

## Additional information

**How to cite this article:** Nestola, F. *et al*. Mineral inclusions in diamonds may be synchronous but not syngenetic. *Nat. Commun.* **8,** 14168 doi: 10.1038/ncomms14168 (2017).

**Publisher's note**: Springer Nature remains neutral with regard to jurisdictional claims in published maps and institutional affiliations.

## Figures and Tables

**Table 1:** Crystallographic orientations for diamond, its two inclusions and the clinopyroxene external to the diamond studied in this work.

			
Diamond host	with	Olivine inclusion	Random orientation
Diamond host	with	Clinopyroxene inclusion	Random orientation
Olivine inclusion	with	Clinopyroxene inclusion	Random orientation
Clinopyroxene inclusion	with	External clinopyroxene	Identical orientation
			
*Reciprocal crystallographic orientation for 100, 010 and 001 axes for the two clinopyroxenes*			
1 0 0	with	1 0 0	0.8°
0 1 0	with	0 1 0	0.6°
0 0 1	with	0 0 1	1.6°

The relative orientations reported were calculated using the software OrientXplot^48^.
